# Indirect Genetic Effects and Housing Conditions in Relation to Aggressive Behaviour in Pigs

**DOI:** 10.1371/journal.pone.0065136

**Published:** 2013-06-06

**Authors:** Irene Camerlink, Simon P. Turner, Piter Bijma, J. Elizabeth Bolhuis

**Affiliations:** 1 Adaptation Physiology Group, Department of Animal Sciences, Wageningen University, Wageningen, The Netherlands; 2 Animal Breeding and Genomics Centre, Department of Animal Sciences, Wageningen University, Wageningen, The Netherlands; 3 SRUC, Edinburgh, United Kingdom; CNRS, Université de Bourgogne, France

## Abstract

Indirect Genetic Effects (IGEs), also known as associative effects, are the heritable effects that an individual has on the phenotype of its social partners. Selection for IGEs has been proposed as a method to reduce harmful behaviours, in particular aggression, in livestock and aquaculture. The mechanisms behind IGEs, however, have rarely been studied. The objective was therefore to assess aggression in pigs which were divergently selected for IGEs on growth (IGEg). In a one generation selection experiment, we studied 480 offspring of pigs (*Sus scrofa*) that were selected for relatively high or low IGEg and housed in homogeneous IGEg groups in either barren or enriched environments. Skin lesion scores, a proxy measure of aggression, and aggressive behaviours were recorded. The two distinct IGEg groups did not differ in number of skin lesions, or in amount of reciprocal fighting, both under stable social conditions and in confrontation with unfamiliar pigs in a 24 h regrouping test. Pigs selected for a positive effect on the growth of their group members, however, performed less non-reciprocal biting and showed considerably less aggression at reunion with familiar group members after they had been separated during a 24 h regrouping test. The enriched environment was associated with more skin lesions but less non-reciprocal biting under stable social conditions. Changes in aggression between pigs selected for IGEg were not influenced by G×E interactions with regard to the level of environmental enrichment. It is likely that selection on IGEg targets a behavioural strategy, rather than a single behavioural trait such as aggressiveness.

## Introduction

The social behaviour of group housed animals is of great importance for their health, welfare, and productivity which may decline due to receipt of harmful social behaviours. Harmful social behaviours, such as aggression, are considered an important problem in commercial livestock farming [1], [2]. Here, we assess the potential of a new breeding method using information on indirect genetic effects, to reduce aggression in pigs.

An Indirect Genetic Effect (IGE), also known as social genetic effect, associative effect or competitive effect, is a heritable effect of an individual on the trait values of its social partners or group mates [3], [4], [5], [6]. The classical example of an IGE is the maternal genetic effect of a mother on trait values of her offspring in mammalian species [5], [7], [8], [9]. Note that the term ‘maternal genetic effect’ does not refer to the effects of genes transmitted by the mother to her offspring, but to the heritable component of the environment that the mother provides to her offspring, e.g., via maternal care behaviour. In other words, with IGEs, the social environment that an individual experiences contains a heritable component [5]. Another well-known case of IGEs in livestock populations occurs in cannibalistic laying hens, where the survival probability of an individual depends on the genotype of its cage mates [6], [10]. IGEs have been studied in several animal species, such as cattle, mice and deer [11], [12], [13], [14], as well as in plants and trees [15], [16], [17], [18].

IGEs can have a profound effect on heritable variation in traits and on response to selection [3], [4]. For example, they can fully remove heritable variation in a trait despite a positive classical heritability [14], [18], and may cause a negative response to positive selection [3], [6]. Hence, when present, IGEs are highly relevant for livestock genetic improvement. By including IGEs in the breeding criteria, both the additive genetic merit of an individual for own performance, the so-called direct genetic effect, and its indirect genetic effect on the performance of its social partners are taken into account. For example, an animal may be a less attractive candidate for selection if it has a high level of individual performance in an economically important trait but shows much aggression towards others, thereby reducing their performance. Due to the potential of IGEs to increase both production and animal welfare, IGEs have become an increasingly important research topic in animal breeding [13].

IGEs are hypothesized to be related to behaviour, and in particular to aggression and competition [4], [6], [12], [19], [20], [21]. However, the actual behaviour of animals with diverging estimated IGEs has rarely been studied. In mice, IGEs have been shown to affect agonistic behaviours [12] whilst in laying hens, selection for IGEs on survival time reduced harmful feather pecking behaviour [22], [23]. In pigs, where IGEs are estimated based on the growth of group members [19], [24], [25], there are indications that pigs with diverging IGEs for growth, though not genetically selected for IGE, differ in the amount of skin lesions [20], [21], which is a commonly used proxy measure of aggression [26], [27].

Aggression is a natural behaviour that contributes to the establishment of dominance relationships, and is most common and intense when unfamiliar conspecifics first meet [26], [28]. Once dominance relationships have been established, aggression is usually limited. In commercial farming, aggression is more likely to escalate, due to management practices such as regrouping unfamiliar animals, and the confined enclosures which may impede retreat after a threat [29]. Aggression is considered a problem for animal welfare and production [2], [26], [30]. Aggressiveness is moderately heritable and can be genetically selected against [2], [31], but phenotyping behavioural traits or their proxy measures is time consuming. Genetic selection for IGEs on growth does not require additional phenotyping and, moreover, targets social interactions as a whole rather than a single behaviour. Genetic selection for IGEs has therefore been proposed as a potential method to improve group production and to reduce harmful behaviours in livestock [2], [13].

In commercial pig farming, pigs are regrouped with unfamiliar pigs as standard management practice, with intense aggression as a result. For several weeks after regrouping, pigs may have an impaired immune response and reduced growth [26], [32], [33], [34]. The level of aggression may vary among environments, as has been shown in amongst others, humans [35], mice [36], fish [37], and pigs [38]. In pigs for example, the availability of bedding substrate suitable for rooting and chewing, such as long-stemmed straw, has been shown to crucially affect behaviour (reviewed by [39]) and mood [40], [41]. Moreover, the expression of a genetic disposition for aggressiveness may largely depend on the environment (reviewed by [42]). To assess whether the outcome of selection for IGEs differs within different environments, the effect of environmental conditions and the extent of genotype by environment interactions should be estimated.

This study therefore investigated whether pigs selected for either high or low IGE on growth, and housed in either a conventional barren pen or a straw-enriched pen, show differences in aggression under regrouping situations and stable social conditions. The hypothesis that pigs selected for high IGE for growth would show less aggression towards group members was assessed by observations on skin lesion scores and aggressive behaviours in pigs divergently selected for IGE on growth.

## Materials and Methods

### Ethics

This study was carried out in strict accordance with the recommendations in the European Guidelines for accommodation and care of animals. The protocol was approved by the Institutional Animal Care and Use Committee of Wageningen University (Protocol Number: 2010055f).

### Indirect Genetic Effects

This section briefly summarizes the theory on IGEs. In quantitative genetics, phenotypic trait values (*P*) in the absence of IGEs are usually modelled as the sum of a heritable component, the breeding value (A), and a residual component, the environment (E); *P* = *A*+*E* [43]. When individuals affect each other’s trait values, this model has to be extended with IGEs [3],
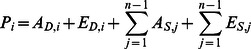
where *i* denotes the focal individual, *j* one of its *n*-1 group mates, *A* denotes heritable effects (also known as breeding values), subscript *D* denotes direct effects and subscript *S* denotes indirect effects. Hence, with IGEs the trait value of an individual is the sum of its own direct genetic effect, 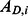, the sum of the IGEs of all its group mates, 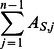, and a non-heritable component, 
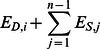
. With this model, response to selection is determined by the change in the so-called total breeding value (*A_T_)* [4], [44], 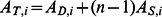, where *n* –1 is the number of group mates excluding the individual itself. Thus, *A_T,i_* represents for each individual the effect of its genes on its own phenotype, plus the effects of its genes on the phenotypes of its group mates.

### Indirect Genetic Effect (IGE) Estimation

Estimated breeding values (EBV) for IGEs were based on growth rate during the finishing phase (from app. 25–110 kg), here abbreviated as IGEg. EBVs were estimated using Best Linear Unbiased Prediction and a so-called animal model [45]. Following [6], [46], [47], the animal model included both the direct effect of the individual, the IGEg of each of its group mates, and a random group effect. Full details of the model are given in [25].

Subsequently, the dams and sires with the most extreme high and low IGEg of the available population were selected to create a F1 population, see Table 1. Dams (Topigs-20 sows: sow line of Great Yorkshire × Dutch Landrace) were selected out of in total 120 sows from the TOPIGS experimental farm. Sires (Tempo boars: commercial synthetic boar line with Great Yorkshire genetic background) were selected from in total 532 TOPIGS AI boars. The contrast for estimated IGEg between the high and low selected offspring was on average 3.6 g/day (Table 1). Average accuracy of the estimated IGEg of the sires was 0.63. Sires and dams were selected so that the average estimated direct genetic effect was similar for both offspring groups (High: 11±2 g ADG; Low: 8±2 g ADG; P = 0.36). After weaning, offspring were housed in groups of six (see section Animals and housing). The IGE contrast, therefore, corresponds to an expected growth difference of (6−1) · 3.6 = 18 g/day [47]. Theoretically, this would mean an expected difference of 2.9 kg in body weight between the pigs from the high and low group at the end of the 160-days trial (at ∼110 kg). Power calculations, taking into account both the uncertainty in the genetic selection differential and the additional noise in the observed response to selection due to non-genetic effects (the *E*-terms in the above expression for *P_i_*), indicated that this response was expected to be significant (*P*<0.05).

**Table 1:** Selection of animals based on estimated IGEg contrast.

	High IGEg		Low IGEg		Contrast (g/day)
	N	Est. IGEg*	N	Est. IGEg	
Selected sires	13	4.36±0.1	11	−1.65±0.1	6.01
Selected dams	34	−0.35±0.05	31	−1.66±0.05	1.31
Offspring	240	2.00±0.6	240	−1.62±0.5	3.62

* Estimated Indirect Genetic Effect for growth rate in grams per day.

### Animals and Housing

A total of 480 offspring were studied over five batches of 96 piglets each. Piglets were born in conventional farrowing crates (TOPIGS experimental farm, Beilen, The Netherlands). Tails and teeth were kept intact, but male piglets were castrated at three days of age. Cross fostering was applied only if litter sizes exceeded 14 piglets, and always within the same IGE group. At approximately 14 days of age, piglets were subjected to the backtest to assess their coping style [48], [49]. Hereby a piglet is placed in a supine position for 60 s whereby its response is indicative of its behavioural strategy. At 26 days of age, piglets were weaned and a maximum of eight (non-cross fostered) piglets per sow were selected. Selection was based on general health, sex, and backtest classification. At weaning, the average weight did not significantly differ between the high and low IGE group. Selected piglets were transported to experimental farm De Haar (Wageningen, The Netherlands). During transportation, all litters were kept separately to avoid aggression. Transportation did not lead to notable skin lesions.

From weaning on, a 2×2 experimental arrangement was applied with IGE (low vs. high) and housing conditions (barren vs. enriched) as factors at the pen level. Within each batch, pigs were housed in 16 pens of six individuals each, giving a total of 80 pens.

On arrival at the farm, each pig was placed immediately in a pen with five unfamiliar pigs. Each pig was identified by a spray marked number on the back, which was refreshed before tests and observations. Group composition was within pen balanced for sex (1∶1) and backtest classification (1∶3 pro-active to re-active coping style, according to the distribution of the whole tested population). The distinct IGE groups were never mixed. Half of the pigs from each IGE group, and half of the selected piglets from each sow, were allocated to a barren pen, and the other half to an enriched pen.

Barren pens had a floor which was half solid concrete and half slatted whilst enriched pens had a solid floor with a deep litter bedding of sawdust and straw. All pens had a space allowance of ∼1.0 m^2^/pig, and contained a metal chain with galvanized ball (75 mm diameter). Dry pelleted commercial feed was offered *ad libitum* from a single space feeder. Water was continuously available from a single nipple drinker per pen. Lights were on from 7∶00 till 19∶00 h. From week eight onwards, all pens received a handful of sawdust per day and a jute sack attached to the wall to reduce damaging tail biting behaviour. Pigs were housed in these pens from weaning until slaughter at 23 weeks of age. Due to diverse health reasons, 18 high IGE pigs and 11 low IGE pigs were removed from the experiment.

### Tests and Observations

The timeframe in which the tests and observations were carried out are presented in Table 2. Data on tests and skin lesions were recorded by a single observer, who was unaware of the IGE group of the pigs. Live behavioural observations were performed by several observers who were unaware of the IGE group of the pigs. These observers were trained beforehand to score in the same way and were balanced across treatment groups.

**Table 2:** Tests and observations by week of age (w).

Age	Test	Behavioural observations	Skin lesions
2 w	Backtest		
4 w	Weaning (d 26)	Scan sampling*(d 27 and d 29)	4 h before weaning24 h after weaning
5 w		Scan sampling	
8 w		Scan sampling	
9 w	Regrouping test (24 h)	Scan samplingContinuous observation (video)	1 h before regrouping24 h after regrouping
10 w	Response to weighing	Scan sampling	
16 w		Scan sampling	
18 w			9 w after regrouping
21 w		Scan sampling	

Pigs (n = 480) were slaughtered at 23 w of age.

* 2-min Instantaneous scan sampling, generally for 6 h per day of observation per pig.

### Regrouping Test

In commercial farms, pigs are usually relocated and regrouped at around nine weeks of age. To simulate this situation, pigs of nine weeks of age were regrouped for 24 h within IGE group and housing condition. The (temporary) new group consisted of three unfamiliar pairs of pigs and within each pair was balanced for sex (1∶1) and within group was balanced for backtest classification (1∶3 pro-active to re-active coping style). None of the pigs in the newly composed groups were full-sibs. Pigs were relocated, within 15 min, into a pen that was unfamiliar to all pigs. Pigs were kept in the new group composition for 24 h, after which they were relocated to their initial pens and reunited with their original pen mates. Behaviour was video recorded from 2 h before the regrouping test until 48 h after the start of the test.

### Skin Lesion Scores

Skin lesions were counted as the number of lesions by body region, following the procedure of [27]. Body regions were front (head, neck, shoulders and front legs), middle (flanks and back), and rear (rump, hind legs and tail). For each body region, a differentiation was made between superficial and deep skin lesions. Deep skin lesions were lesions where skin was broken, showing signs of haemorrhage. Skin lesions were counted before and after encounters with unfamiliar pigs, see Table 2. For the skin lesion score 24 h after regrouping, a cell counter was used to facilitate the counting of lesions.

### Live Behavioural Observations

Behaviours of individual pigs were recorded on eight days in total, see Table 2. The ethogram included reciprocal fighting, aggressive non-reciprocal biting, head knocks and aggression at the feeder. Aggression at the feeder included all reciprocal fights, aggressive non-reciprocal bites, and head knocks given within <1 m distance from the feeder. All other active behaviours were summed to approximate a general activity level. Behaviour was scored during live observations using 2-min instantaneous scan sampling, for 6 h per day between 8∶00 and 17∶00 h. The Observer 5.0 software package (Noldus Information Technology B.V., Wageningen, The Netherlands) installed on a hand-held computer, was used for behavioural recordings.

### Video Observations after the Regrouping Test

Videos from immediately after the regrouping test, when pigs were reunited with their original pen mates, were analysed for number of aggressive interactions per pen. From the moment that all six pigs had returned to their home pen until 30 min thereafter, the number of reciprocal fights, non-reciprocal (series of) bites, head knocks and fights at the feeder were counted per pen. Reciprocal fights and non-reciprocal series of bites were counted from the start of a series of aggressive interactions until either the end of the fight or series of non-reciprocal bites, or a pause of at least 3 s.

### Response to Handling at Weighing

Response to handling at weighing previously showed a positive genetic correlation with aggression (r_g_ 0.41–0.60) [50], and was therefore included in this study. At 10 weeks of age pigs were weighed and the response of the pigs to handling at weighing was scored. This was the first time that the pigs experienced a weigh crate. Behaviour during weighing was scored as previously described [50]. Briefly, each pig received three scores: a 1–5 score for the ease of entering the crate, a 1–3 score for movements in the crate and, a 1–3 score for ease of leaving the crate. The lower the score, the more resistance the pig showed to handling or being in the crate. The number of vocalizations was recorded from entering the crate until the moment the pig left the crate.

### Data Analysis

Statistical analyses were performed using SAS (SAS 9.2, Institute Inc.). Residuals of the response variables were checked for normality.

To test whether the skin lesion score differed between IGE groups and housing conditions, pre-mixing skin lesion scores were subtracted from the number of skin lesions after regrouping. Negative values were set to zero. The number of skin lesions on the body as a whole (sum front, middle and rear) was square root transformed to achieve a normal distribution, and analysed in a mixed model (Mixed Procedure), with IGE group, housing condition, the interaction between IGE group and housing condition, sex and batch as fixed effects and pen nested within IGE group, housing condition and batch as random effect. Scores on the separate body parts were not normally distributed after transformation and were analysed as described above, but with the raw data in a generalized linear model with Poisson distribution (Glimmix Procedure).

Scan samples from the live behavioural observations were expressed as the proportion of total observation time spent on a behaviour and were analysed separately for each observation day. To obtain a normal distribution, behaviours were arcsine square root transformed. Effects of IGE group and housing condition on aggressive behaviour were analysed in a mixed model as described above (Mixed Procedure). Including general activity in the model did not lead to considerable changes in the reported P values and was therefore omitted from the model. The number of aggressive interactions as observed from video footage was recorded on a pen level and therefore analysed with a general linear model (GLM Procedure) including the effects of IGE group, housing condition and batch.

Response to handling at weighing was tested for differences between IGE groups and housing conditions. Data on batch 3 had to be omitted due to technical problems with the weigh crate. Scores on entering the crate were analysed with a mixed model as described above for skin lesions (Mixed Procedure). For movements in the crate and leaving the crate, score 1 was combined with score 2, since only 3 pigs had score 1 for movements in the crate, and 15 pigs had score 1 for leaving the crate. Scores for movement in the crate and on leaving the crate were therefore analysed using a generalized mixed model with a binary distribution and a logit link function (Glimmix Procedure).

Data are presented as (untransformed) means ± SEM.

## Results

### Skin Lesions

The number of skin lesions did not significantly differ between high IGE and low IGE pigs at any scoring time or on any body region (Table 3). Intensity of the lesions (superficial or deep) did not significantly differ between high and low IGE pigs (P = 0.54). Pigs housed in enriched pens had higher skin lesion scores at all sampling points under stable social conditions and at 24 h after the regrouping test on the middle and rear of the body, and had also more deep lesions on the total body (Barren: 6.8±0.4; Enriched: 8.1±0.4 lesions; P = 0.004). The interaction between IGE group and housing condition had no significant effect on the lesion scores (P = 0.87). Female pigs had more skin lesions on all scoring days (mean total lesion score over all scoring days: females 139±5 vs. male 124±5; P = 0.05), but this effect differed by body region on each recording day.

**Table 3:** Number of skin lesions for high and low IGE pigs in barren and enriched housing, for each body region, by week of age (w) with weaning at 4 w of age and the regrouping test (RT) at 9 w.

		High IGE		Low IGE		P-value	
Age	Region	Barren	Enriched	Barren	Enriched	IGE	HC
4 w	Front	17.8±2.4	22.2±2.4	20.4±2.4	23.0±2.4	0.16	0.18
	Middle	6.9±1.1	9.9±1.1	8.5±1.1	10.1±1.1	0.41	0.14
	Rear	4.5±0.8	5.1±0.8	4.3±0.8	6.1±0.8	0.61	0.16
9 w	Front	2.8±0.4	4.2±0.4	3.0±0.4	4.9±0.4	0.18	<0.001
	Middle	1.9±0.4	4.7±0.4	1.9±0.4	3.6±0.4	0.66	<0.001
	Rear	0.7±0.2	1.7±0.2	0.6±0.2	1.5±0.2	0.64	<0.001
9 w RT	Front	34.6±3.0	41.9±3.0	33.4±3.0	35.4±3.0	0.96	0.64
	Middle	25.3±3.1	35.4±3.1	27.6±3.0	31.4±3.0	0.75	0.23
	Rear	11.1±1.8	15.5±1.8	14.5±1.8	17.5±1.8	0.07	0.17
16 w	Front	3.3±0.4	5.1±0.4	3.3±0.4	5.4±0.4	0.79	<0.001
	Middle	1.9±0.4	3.7±0.4	2.1±0.4	3.9±0.4	0.87	<0.001
	Rear	1.5±0.3	2.7±0.3	1.5±0.3	2.9±0.3	0.86	<0.001

P-values are given for the difference between IGE groups (IGE) and housing conditions (HC).

### Behavioural Observations

**Figure 1:**
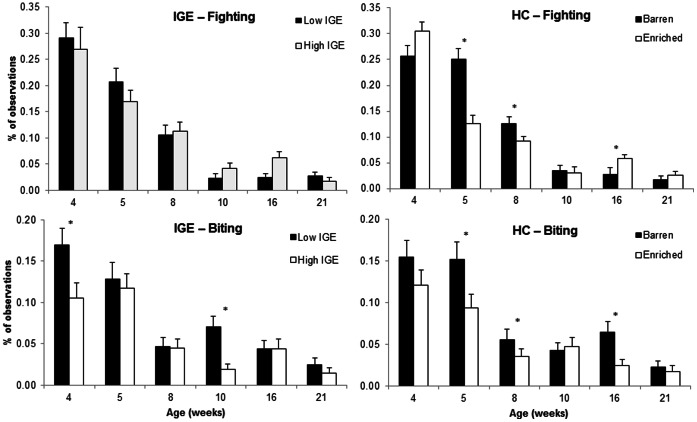
Aggressive behaviour from life observations. Percentage of observation spent on reciprocal fighting and non-reciprocal biting for IGE group (low and high IGE) and housing condition (HC, barren and enriched) over weeks of age outside regrouping situations (4 w and 9 w). Significant differences are indicated by *P<0.05.

High IGE pigs showed less non-reciprocal biting behaviour than low IGE pigs in week 4 (High: 0.10±0.02; Low: 0.17±0.02% of observations; P = 0.006), three days after weaning, and week 10 (High: 0.02±0.01; Low: 0.07±0.01; P<0.001), seven days after the regrouping test (Figure 1). There was no significant difference between high and low IGE pigs in the number of reciprocal fights, except for week 16, when high IGE pigs in enriched pens fought more (High: 0.06±0.01; Low: 0.02±0.01; P = 0.02). The IGE groups did not differ in the amount of head knocks (P = 0.32) or fights at the feeder (P = 0.62). Housing conditions influenced the amount of aggression during the weeks after the regrouping situations, whereby pigs in barren pens showed more biting in week 5 (Barren: 0.15±0.02; Enriched: 0.09±0.02% of observations; P = 0.03) and week 16 (Barren: 0.06±0.01; Enriched: 0.02±0.01; P = 0.03; Figure 1). Pigs in barren pens also showed more reciprocal fighting in week 5 (Barren: 0.2±0.03; Enriched: 0.1±0.03; P = 0.008) and week 8 (Barren: 0.14±0.02; Enriched: 0.08±0.02; P = 0.04; Figure 1). There tended to be an interaction between IGE group and housing condition for non-reciprocal biting in week 4 (P = 0.06), due to a higher in amount of biting in low IGE pigs in barren pens as compared to high IGE pigs in enriched pens (High-E: 0.11±0.02; Low-B: 0.21±0.02% of observations; P = 0.004). An opposite interaction tended to exist for fighting in week 16, where high IGE pigs in enriched pens fought more than low IGE pigs in barren pens (High-E: 0.09±0.01; Low-B: 0.02±0.01; P = 0.004).

On the day after weaning, the amount of non-reciprocal biting and reciprocal fighting did not differ between IGE groups (Biting: P = 0.97; Fighting: P = 0.63) or between housing conditions (Biting: P = 0.78; Fighting: P = 0.12). During the regrouping test at 9 weeks of age, the amount of non-reciprocal biting and reciprocal fighting did not differ between IGE groups (Biting: P = 0.98; Fighting: P = 0.14). Pigs in enriched pens showed more biting during the regrouping test (Barren: 0.32±0.04; Enriched: 0.46±0.05% of observations; P = 0.02), but not more reciprocal fighting (P = 0.35). Female pigs showed more aggressive behaviour during all observation days (Females: 0.42±0.02; Males: 0.35±0.02; P = 0.02), except for weaning and the first week after weaning (weeks 4 and 5). In week 5, males showed more aggression (Females: 0.53±0.04; Males: 0.64±0.04; P = 0.03).

### Aggression at Reunion

**Figure 2:**
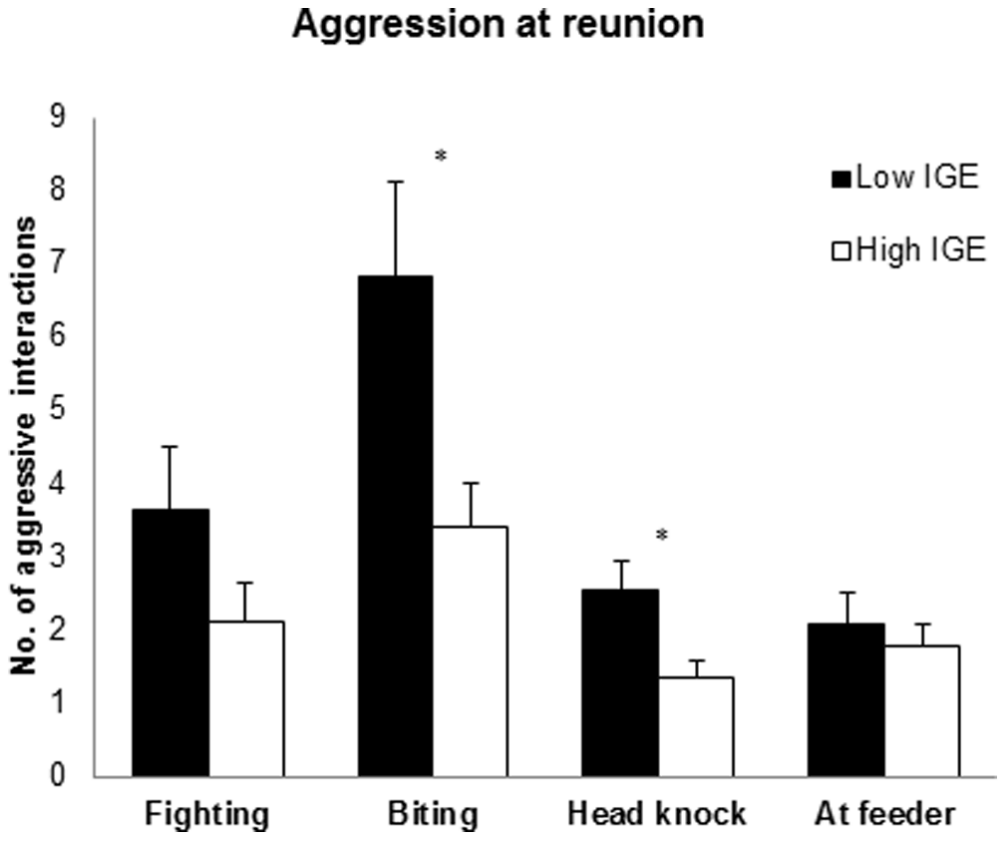
Aggression at reunion. Frequency of aggressive interactions within low and high IGE pens during the 30 min after reunion by type of aggressive behaviour. Aggression at feeder includes fighting, biting and head knocks given within <1 m of the feeder. Significant differences are indicated by *P<0.05.

The distinct IGE groups showed considerable behavioural differences upon reunion with familiar group members after having been separated for 24 h during the regrouping test. In the first 30 min after reunion, pigs from high IGE pens had on average 8.0±1.8 aggressive interactions, whereas pigs from low IGE pens had 15.7±1.8 aggressive interactions (P = 0.004). In high IGE pens, there was less non-reciprocal biting (High: 3.4±0.6; Low: 6.8±1.3 occurrences in 30 minutes; P = 0.008) and there were fewer head knocks (High: 1.4±0.2; Low: 2.6±0.4 occurrences in 30 minutes; P = 0.02; Figure 2). In 9 out of 60 cases low IGE pens had more than 20 aggressive interactions (range 0–49), while none of the high IGE pens reached this number of encounters (range 0–18). There was no effect of housing condition on the amount of aggression shown (P = 0.85), nor an interaction between IGE group and housing condition (P = 0.44).

### Response to Handling at Weighing

In general, pigs entered the weigh crate after little encouragement by an animal handler. High IGE pigs entered the weigh crate more easily than low IGE pigs (High: score 3.8±0.1; Low: score 3.6±0.1; P = 0.03). Pigs housed in enriched pens also entered the crate more easily (Enriched: score 3.8±0.1; Barren: score 3.6±0.1; P = 0.04). Pigs mostly stood still in the crate (score 2.8±0.02) and left the crate after some encouragement of the animal handler (score 2.4±0.04). There was no significant effect of IGE group on movements in the crate (P = 0.75) or resistance to leaving the crate (P = 0.79), nor for housing conditions (P = 0.97 for movements on the crate; P = 0.50 for leaving the crate). There was no interaction between IGE group and housing condition on any of the measurements for response to weighing (overall P = 0.30).

## Discussion

We hypothesized, as aggression may reduce growth [32], [33] that animals selected for high IGE for growth (IGEg) would be less aggressive than animals selected for low IGEg. In this study, high IGE and low IGE pigs did not differ in number of skin lesions or in time spent fighting. High IGE pigs, however, performed less biting and showed considerably less aggression at reunion with familiar group members after they had been separated during a 24 h regrouping test at 8 w of age. Pigs in enriched housing conditions had more skin lesions but showed less aggressive behaviour. There was no significant interaction between IGE group and housing condition. It therefore seems unlikely that possible changes in aggressive behaviour of pigs selected for IGEg would differ between barren and enriched pens.

### IGEg and Skin Lesions

Previous studies on pigs with estimated breeding values for IGE on growth showed that high IGE pigs had more skin lesions on the front of the body after regrouping, but had fewer lesions under stable social conditions in the weeks after regrouping [20], [21]. Skin lesions on the front of the body are typically received during reciprocal fighting and those on the rear of the body indicate that the pig has been bullied [51]. It was therefore suggested that high IGE pigs are more competent at establishing dominance relationships. In the current study, skin lesion scores did not significantly differ between the IGE groups. This discrepancy with the previous study might be due to differences in the experimental design. This was the first study where a large number of pigs were selected on extremes of estimated IGEg and housed in distinct IGE groups, whereas in previous studies pigs were randomly grouped together without prior knowledge of their IGEg [21]. Based on skin lesion scores alone, we could not confirm that high IGE pigs would show less aggression, or would be better able to establish dominance relationships, than low IGE pigs.

### IGEg and Aggressive Behaviour

Behavioural observations did reveal differences in aggression between high and low IGE pigs. The main result was that high IGE pigs showed considerably less aggression in the first 30 min after reunion with familiar pen mates after 24 h exposure to unfamiliar pigs in a regrouping test. We expected to see a difference within the 24 h of regrouping because this period is often studied for the intense aggression that occurs in this timeframe. In commercial farming, animals are not reunited after regrouping, but this unexpected finding may provide important information on behavioural strategies that may change in animals selected for IGEg. We here outline three potential mechanisms.

One hypothesis for why high IGE pigs fought less when they were reunited with familiar pen mates could be that dominance relationships were more stable in high IGE groups beforehand, or that high IGE pigs apply a different dominance style [52] and, that therefore, high IGE pigs could re-establish their dominance relationships with less aggression at reunion. High IGE pigs showed less non-reciprocal biting in both weeks after a regrouping situation, which would be in line with this hypothesis [20], [21]. Also when aggression is limited, as non-reciprocal biting in the weeks after regrouping occurred on an average of only 0.4% of the behavioural scans, dominance relationships may still have an effect on health and stress levels [53], [54]. Although instantaneous scan sampling may underestimate the amount of short lived behaviours such as non-reciprocal biting [55], and in reality the amount of aggressive interactions would be higher as observed from scan samples, we are cautious about drawing conclusions based upon this difference in IGE groups.

Another hypothesis could be that low IGE pigs experience more stress after social interruptions such as regrouping, or that they were more inclined to direct their stress or frustration towards pen mates than high IGE pigs given the same level of stress [33], [56]. Differences between the IGE groups became apparent after weaning and regrouping, expressed in non-reciprocal biting. Non-reciprocal biting is also referred to as bullying behaviour [50], but may also have an important function in stress-induced aggression as biting may suppress the release of stress-induced noradrenaline and ulcer formation [57], [58]. Potentially, low IGEg pigs evaluate a social interruption differently or are more likely to direct their response towards group members.

A third hypothesis could be that high IGE animals are better able to recognize or remember their original group members. During stressful situations, social recognition or social memory may be impaired [59], [60], which may increase aggression [61], [62], [63]. It is possible that high IGE animals have better social recognition, or are better able to cope with stressful situations as the differences in aggressive behaviour between IGE groups were in all cases present in the week after a stressful regrouping event.

From both this study and the studies of [20] and [21] it seems that selection for IGEg does affect aggression related behaviour in pigs. Previous studies showed that selection for IGEs influenced aggression in mice [12], and influenced feather pecking behaviour in laying hens [23]. It is possible that selection for IGEg affects a range of behavioural traits of which aggression is one. This range of traits may for example affect the way in which dominance relationships are established [20], [21] or social cohesion is maintained [64]. The fact that high IGE pigs entered the weigh crate more easily shows that selection for IGEg changes more than the expression of aggression alone. Difficulty with entering the crate might reflect an aggressive temperament [50], [65], but may also reflect, for example, stress susceptibility, fear of humans or novel situations, or sociability by moving towards or away from group members.

### Housing Conditions and Aggression

The environment can contribute to the expression of aggression, irrespective of the genetic merit of an individual for aggressiveness [42], [66]. The direction in which the environment affects aggression, however, appears to differ both between and within species [36], [38], [66]. The same holds for pigs, where enriched pens may lead to less aggression [67], no difference in aggression [68], [69], [70], or more aggression [71], [72] as compared to barren pens of equal size. In the current study, pigs in straw-enriched pens had more skin lesions under stable social conditions, but showed less non-reciprocal biting than pigs in barren pens. Though the number of skin lesions may be underestimated due to skin dirtiness [72], [73], [74], as pigs in the barren pens had a more dirty skin (unpublished results), lesions were clearly visible and were scored when the observer was in close proximity to the animal.

Animals in an enriched environment may have more injuries, like skin lesions [38], [68], due to higher activity levels or due to competition over resources [75], such as fresh substrate or a dry lying area [76]. Skin lesions under stable social conditions may have also been caused by play behaviour or comfort behaviour, like scratching, which occurred more in enriched environments (Camerlink et al., in prep.) [58]. When skin lesions, which are considered as a heritable trait [2], [27], are used to reduce aggression through direct breeding, the likelihood of increased skin lesions due to an enriched environment should be taken into account. During regrouping situations, housing conditions may have less effect on the number of skin lesions as animals will fight regardless of their environment when they first meet an unfamiliar conspecific [28], and lesion scores may better reflect the amount of aggressive interactions.

### Selection for IGEg

At present, very little is known of the mechanisms underlying IGEs for growth rate in pigs. When behaviours underlie IGEg, differences in behaviour may be a precursor to differences in growth. If that is true, one generation of selection might not be sufficient to detect differences in growth between groups despite the *a priori* power calculations which suggested a sufficient contrast. For the trait under selection, i.e. growth rate, indeed no phenotypic differences were found between both IGE groups (Camerlink et al., in prep.). Differences in behaviour, however, may already be present after one generation of selection. Differences in aggressive behaviour between the IGE groups in this study were small and point to a difference in behavioural strategy rather than aggressiveness per se. Similar indications come from a selection experiment in laying hens selected based on total breeding value for survival time, which showed distinct patterns in harmful pecking behaviour [23] while the differences in pecking-related mortality were less clear (personal communication ED Ellen). It would be worthwhile to investigate these behavioural differences after multiple generations of selection for IGEg.

### Conclusion

This is the first study where a large number of pigs was selected and grouped based on IGE for growth (IGEg). Selection for high IGEg did not affect the major aggression parameters in pigs, namely skin lesion scores and fighting during regrouping. The results show, however, that this first stage of selection considerably reduced aggression at reunion with familiar group members and gave a small reduction in non-reciprocal biting in the weeks after regrouping. Changes in aggressive behaviour as a consequence of selection for IGEg do not seem to be influenced by a G×E interaction with regard to the level of environmental enrichment. Aggression may be one facet of the possible ways in which group housed animals may influence each other’s growth. If IGEg are included in the breeding criteria it would be important to consider the possible changes in behaviour over generations.
